# Fringe Projection Profilometry in Production Metrology: A Multi-Scale Comparison in Sheet-Bulk Metal Forming

**DOI:** 10.3390/s21072389

**Published:** 2021-03-30

**Authors:** Lennart Hinz, Sebastian Metzner, Philipp Müller, Robert Schulte, Hans-Bernward Besserer, Steffen Wackenrohr, Christopher Sauer, Markus Kästner, Tino Hausotte, Sven Hübner, Florian Nürnberger, Benjamin Schleich, Bernd-Arno Behrens, Sandro Wartzack, Marion Merklein, Eduard Reithmeier

**Affiliations:** 1 Institute of Measurement and Automatic Control, Leibniz Universität Hannover (LUH), 30823 Garbsen, Germany; markus.kaestner@imr.uni-hannover.de (M.K.); eduard.reithmeier@imr.uni-hannover.de (E.R.); 2 Institute of Manufacturing Metrology, Friedrich-Alexander-Universität Erlangen-Nürnberg (FAU), 91052 Erlangen, Germany; sebastian.metzner@fmt.fau.de (S.M.); tino.hausotte@fmt.fau.de (T.H.); 3 Institute of Forming Technology and Machines, Leibniz Universität Hannover (LUH), 30823 Garbsen, Germany; mueller@ifum.uni-hannover.de (P.M.); huebner@ifum.uni-hannover.de (S.H.); behrens@ifum.uni-hannover.de (B.-A.B.); 4 Institute of Manufacturing Technology, Friedrich-Alexander-Universität Erlangen-Nürnberg (FAU), 91058 Erlangen, Germany; robert.schulte@fau.de (R.S.); marion.merklein@fau.de (M.M.); 5 Institut für Werkstoffkunde (Materials Science), Leibniz Universität Hannover (LUH), 30823 Garbsen, Germany; besserer@iw.uni-hannover.de (H.-B.B.); wackenrohr@iw.uni-hannover.de (S.W.); nuernberger@iw.uni-hannover.de (F.N.); 6 Engineering Design, Friedrich-Alexander-Universität Erlangen-Nürnberg (FAU), 91058 Erlangen, Germany; sauer@mfk.fau.de (C.S.); schleich@mfk.fau.de (B.S.); wartzack@mfk.fau.de (S.W.)

**Keywords:** fringe projection, production metrology, sheet-bulk metal forming, coordinate metrology

## Abstract

Fringe projection profilometry in combination with other optical measuring technologies has established itself over the last decades as an essential complement to conventional, tactile measuring devices. The non-contact, holistic reconstruction of complex geometries within fractions of a second in conjunction with the lightweight and transportable sensor design open up many fields of application in production metrology. Furthermore, triangulation-based measuring principles feature good scalability, which has led to 3D scanners for various scale ranges. Innovative and modern production processes, such as sheet-bulk metal forming, thus, utilize fringe projection profilometry in many respects to monitor the process, quantify possible wear and improve production technology. Therefore, it is essential to identify the appropriate 3D scanner for each application and to properly evaluate the acquired data. Through precise knowledge of the measurement volume and the relative uncertainty with respect to the specimen and scanner position, adapted measurement strategies and integrated production concepts can be realized. Although there are extensive industrial standards and guidelines for the quantification of sensor performance, evaluation and tolerancing is mainly global and can, therefore, neither provide assistance in the correct, application-specific positioning and alignment of the sensor nor reflect the local characteristics within the measuring volume. Therefore, this article compares fringe projection systems across various scale ranges by positioning and scanning a calibrated sphere in a high resolution grid.

## 1. Introduction

**Figure 1:**
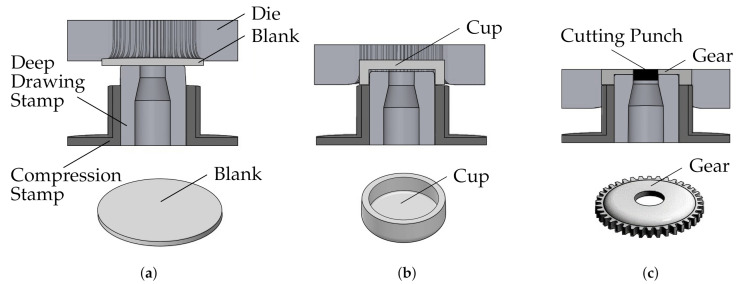
Multi-stage sheet-bulk metal forming (SBMF) process for the production of components with rotationally symmetrical external gearing [5]. (**a**) Initial state; (**b**) Deep drawing; (**c**) Compression and shear cut.

**Figure 2:**
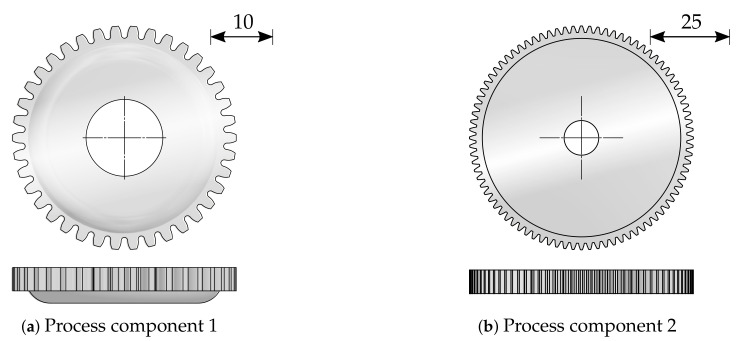
Process components made by sheet-bulk metal forming (SBMF).

The demand for new resource-saving production methods drives the development of new technologies [1]. Sheet-bulk metal forming (SBMF) combines the advantages of sheet metal forming with the advantages of bulk forming. Thus, SBMF enables the production of complex functional components with secondary forming elements made of thin sheet [2]. Therefore, components in which production would otherwise be dependent on other production methods, such as machining, can be manufactured by the use of forming technology [3]. This brings advantages in terms of shorter process times, increased strain hardening, and a near-net shape production [4]. Within the scope of the transregional collaborative research center 73 (TCRC 73) various applications and research challenges of this novel process technology were explored. This has resulted in various production processes in different sub-projects at different locations. SBMF processes combine numerous methods of forming technology. As an example of such a process, a stage sequence for the production of a component with internal and external gearing is shown in Figure 1 [5]. In this process, a cup is first deep-drawn from a 4 mm wide round blank. The semi-finished material used is a low-alloy steel *DC04* (St14) with material number 1.0338 in a nominal sheet thickness of 2 mm which is supplied by Salzgitter Flachstahl GmbH (Salzgitter, Germany). This material has a purely ferritic microstructure and is often used in the cold-rolled condition for the forming of inner and outer car body components, but also for elements in the household appliance industry. *DC04* has a tensile strength of 270 to 350 MPa. Afterwards an external gearing is produced by compressing the cup frame into the gear cavity of a tool die. According to Figure 1c, a hole is shear-cut simultaneously in the center of the cup. In subsequent steps, an internal gearing is ironed and calibrated. This process serves to explore and extend the limits of the technology SBMF. Achievable geometrical, topographical, and mechanical properties for the production of process component 1 were studied and analyzed in detail by Koch [6]. Additionally, tailored surfaces for the specific friction adjustment on tool surfaces were installed [7], and the influence of oscillating tool elements on increasing the material flow [5] and on improving the surface quality [8] was investigated. The produced gear component with a total of 38 teeth is shown in Figure 2a.

**Figure 3:**
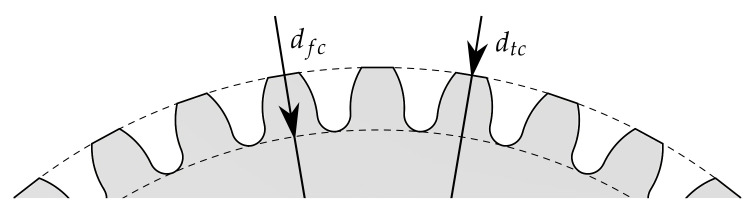
Geometric parameters on the formed specimen parts.

The tip circle diameter $d_{tc}$ (see Figure 3) is 36.4 mm, while the foot circle diameter $d_{fc}$ is 32.4 mm. To qualify and evaluate the measurement methods, a second process is used to manufacture geared components with larger diameter and larger tooth height. This process combines a deep drawing and an upsetting operation in order to manufacture an externally geared cup with 84 teeth [9]. According to Figure 2b, $d_{tc}$ is 85.4 mm, while $d_{fc}$ is 80.4 mm. Due to the adapted tool setup, the force-controlled process also proceeds within a single press stroke. The setup consists of four active tools. The drawing die and the upsetting punch are part of the upper tool, and the upsetting plate and the drawing die are part of the lower tool. In the initial position, the blank with an initial diameter of 100 mm and an initial sheet thickness of 2 mm is positioned on the drawing punch. After the blank is clamped by the upsetting punch, the drawing die moves downwards to draw the cup. In the final step, the cup is upset by the upsetting punch under the adjusted maximum press force [10]. As for the previous process, tailored approaches were developed to extend identified process limits. Especially, tailored blanks and tailored surfaces enable enhanced part quality by improved material flow control within the forming process.

## 2. Problem Definition

### 2.1. Scope of the Metrological Problem

In order to optimize the forming processes with regard to shaping and form filling, it is important to precisely measure the generated component geometries. Therefore, conclusions can be drawn about the influence of specific process parameters on the component characteristics. It is necessary to scan the component on different scales. On a macroscopic level, a measurement of coaxiality of cylindrical components may allow the drawing of conclusions about possible inaccuracies in the placement of semi-finished products in the tool system. This enables the assessment of the influences of process-related variables, such as forming force, tool geometry, or lubrication conditions, on the filling of the functional elements. Furthermore, the measurement data make it possible to determine the influence of semi-finished product parameters, such as initial geometry or material selection, on the final component characteristics. On a microscopic level, a scanning of the surface roughness of the components also provides information about various forming parameters that can be set in the process or by the semi-finished product.

Next to the optimization of the forming process, a further objective is the characterization of the in-service properties of the components based on precise knowledge of the geometry especially of the functional elements. Since the application of SBMF-processes aims at a near-net-shape production without further post-processing, the remaining mold filling errors, eccentricities or surface defects, such as notches, represent a relevant influence on the component properties. While mold filling defects reduce the effective component cross section, notches represent locations of a potential stress concentration during operation of the components and, thus, affect their fatigue life [11,12]. In addition, eccentricities of the components can lead to high-frequency fluctuations in torque during operation of the gears [13]. In order to estimate the stresses during service correctly and calculate them based on common Finite element method (FEM) simulations, the precise geometries of the components are required [14].

Possible surface defects from the forming process can also cause a notch effect, which can significantly reduce the fatigue strength due to the stress increase in this components’ area. Under continuous cyclic loading, fatigue cracks can arise here which ultimately cause the failure of the component. Fatigue life modeling based on fracture mechanic models is another major research topic that is being explored within the scope of TCRC 73 using the exemplary case of externally toothed components and flange components. In addition to investigations of microstructural damage and its influence on the fatigue behavior [15], reliable data on the components’ geometry is also required to enable a process design that is appropriate for the later service life of the component [16].

In order to accompany the development of this innovative production process in the most effective way, adequate measurement principles for the precise measurement of various process-specific features in different scale ranges are necessary. In addition, it requires defined methods and tools to ensure proper feedback between the measurement technology and the production process and to continuously improve the overall performance of the process. For this purpose an engineering workbench was created, which facilitates the support for the scientists and later product developers in conjunction with the SBMF technology. This engineering workbench is called *SLASSY* (self-learning assistance system) [17]. *SLASSY* enables the storage of expert knowledge about the manufacturing process in its database via prediction models and analysis functions for current parts in design. Therefore, it provides a two-step solution: Step one being the synthesis part, where product developers can design individual SBMF parts via an intelligent product configurator which directly communicates with the Computer aided design (CAD) system currently in use. Step two is the analysis part mentioned above, where the prediction models and analysis function are evaluated on their initial part design from the synthesis step. Both steps can be done iteratively and it is always possible to refine the current part design and make a new analysis afterwards. In the context of this contribution, *SLASSY* is used to store the metrological or experimental results in a knowledgebase via knowledge discovery in databases and data mining methods [18]. The main goal of the application of the workbench is a prediction about the necessary measurements and the manufacturing process, which should be used to achieve the best results. On the one hand, *SLASSY* needs to be fed with the manufacturing process data and on the other hand, it needs to be fed with the measurement data and metadata. Product developers need to have a simple overview of the available technology (measurements, processes) and the results that are possible, with their current part or product in design.

### 2.2. Motivation of the Conducted Experiments and Differentiation from the State of the Art

Over the last few decades, in line with the development of digital camera technology, a wide range of optical-holistic measuring sensors has been introduced to the market as a complement to traditional tactile instruments [19,20]. Especially, the triangulation-based approaches, such as the fringe projection profilometry (FPP), are applied in many areas of production measurement technology and process automation [21]. The sensors can extract millions of data points at short exposure times and with sufficient accuracy. In addition, the systems are lightweight, transportable, and comparatively inexpensive. The measurement technology is well scalable, and the design of the triangulation basis and the imaging optics allow for an application-specific balancing between measurement volume and axial resolution [22].

As outlined, one of the key challenges in the optical, metrological reconstruction of process-specific features at different scale ranges is, in addition to the selection of the appropriate sensor, the positioning and orientation of the specimen within the measuring volume [23]. Since surface reconstruction by active triangulation, i.e., fringe projection can only be performed in the overlapping areas of the viewing cones of camera and projector, a significant influence of the specimen pose on the size and accuracy of the reconstructed surface is likely. The viewing cones are limited by the depth of field (DOF) and the arrangement can also cause quantization effects with respect to the pixel resolution of camera and projector [24]. In combination optical aberrations, such as field curvature, the performance of a sensor is not evenly distributed over the entire measuring volume. The spatial orientation, as well as the reflection properties of the technical surface, affects the reconstruction result in many ways [25]. In addition, marginal regions of the measurement volume may typically be insufficiently weighted and, thus, less accurately reconstructed due to lower feature density with respect to the marker-extraction of the system calibration [26]. Therefore, industrial norms and test methods consider a wide range of influencing factors of optical measurement systems. Especially, for multi-view systems, guideline VDI/VDE 2634-3 [27] provides a comprehensive consideration of various influencing factors. Since with each further system parameter and testing method the experimental complexity increases significantly, investigations are typically carried out with only a few configurations. Therefore, maximum permissible errors as specified in guideline VDI/VDE 2634-2 [28] for optical systems based on area scanning and DIN EN ISO 14253-1 [29] are usually provided for the entire measuring volume. DIN EN ISO 10360-7 [30] and DIN EN ISO 10360-8 [31] introduce an essential supplement for optical surface probing sensors with respect to reconstructed point clouds. Due to the extensive geometric constellations and the random character of the experiments, probing patterns are specified in DIN EN ISO 10360-5 [32]. As a result of the complexity of the test procedures, the various parameters to be determined, the applicability of multiple sensor types, and the combination of various industrial standards, the guideline VDI/VDE 2617-6.2 [33] provides a framework for a consistent application of the previously mentioned standards, taking into account the different optical measurement methods.

Global assessments evaluate the characteristics and configurations of a sensor under defined, comparable conditions with limited effort. A precise estimation of the size and shape of the measurement volume, the possible total area of reconstruction and achievable accuracy depending on the relative pose within the measurement volume is not possible and could not be derived with certainty from the limited scope of the assessments. Furthermore, the different physical interactions cannot be precisely observed and separated, which renders interpretation and applicability of the results and subsequent research more difficult. In addition to the complexity of the test procedures, the test conditions are limited by the maximum permissible cone angle, which is to be specified by the manufacturer. A unbiased, experimental assessment of a sensor’s performance can, thus, only be achieved to some extent. Furthermore, measurement conditions are not considered which may occur in practical applications, especially if applicants lack detailed information on the various characteristics depending on position and orientation within the measurement volume.

In this study, experiments are performed under laboratory conditions with optimal exposure settings on an optically cooperative, calibrated sphere, which basically has no preferred orientation. This eliminates many external influencing factors and also the operation possibilities or configurations are basically reduced to the position of the specimen within the measuring volume. Therefore, a high-resolution positioning grid of the specimen within the measurement volume enables a much more detailed, local analysis of the properties of the respective 3D scanner and ensures a optimal application-specific sensor selection and positioning and represent a methodological complement to state-of-the-art assessments.

Since certain sensor-specific characteristics, such as the metrological structural resolution, cannot be assessed by the experiments on calibrated spheres, and in order to provide a possible application within SBMF, the 3D scanners are compared with each other in a second experiment by reconstructing a tooth of a formed gearwheel.

## 3. 3D Scanner Overview

**Figure 4:**
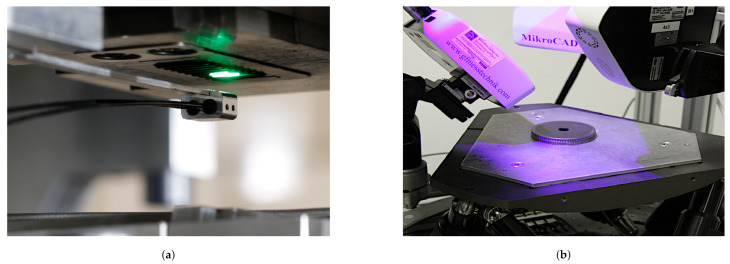
Various applications of fringe projection 3D scanning within the transregional collaborative research center 73 (TCRC 73) at different scale ranges. (**a**) Endoscopic in-situ inspections inside a forming plant (TR 73 Endo 20); (**b**) Multiscale triangulation 3D scanner cluster with high-precision hexapod positioning unit.

Within the scope of the TCRC 73, fringe projection profilometry is utilized in numerous fields of application. Some of the sensors are used individually but also in a cluster of multiple sensors, as shown in Figure 4b. One approach developed within the TCRC 73 is the combination of several 3D scanners in a common coordinate system to create a holistic dataset [34]. Not only the different resolutions of the various fringe projection scanners can applied to different functional elements, but also the combination of datasets with an adjusted registration method with the use of high precision positioning systems is possible [35]. Table 1 shows an overview of various commercial fringe projection 3D scanners applied within the TCRC 73.

In the context of the TCRC 73, research was also conducted on new sensors, which, by combining fringe projection measurement technology with glass fiber bundles, enabled a new class of instruments to be established as a highly compact endoscopic triangulation system [36,37]. This sensor is optimized for robot-guided inspection in narrow spaces due to its design and flexible positioning [38]. Within the TCRC 73, in-situ inspections were carried out during the ongoing forming process to quantify the tool condition between consecutive forming cycles and to predict failure [39]. Such an application is shown in Figure 4a. The endoscopic fringe projection system is based on a phase-coded projection approach [40,41], adopting a heterodyne projection sequence to avoid the influence of crosstalk of individual image fibers within the fiber optic bundle [37]. The subsequent phase unwrapping is based on the work of Creath [42], as well as Servin, Quironga, and Padilla [43]. Camera and projector are calibrated based on the pinhole camera model [44,45], the calibration procedure is mainly based on the work of Heikkilä et al. [46] and the toolbox of Bouguet [47]. A distortion correction follows the approach of Conrady and Brown [48]. For different measurement applications, different sensor head designs have been developed with different working distances [39]. Gradient index rod-lenses supplied by GrinTech GmbH (Jena, Germany) are applied at 10 and 20 mm working distance (wd). The triangulation angle is set to 30° in both cases. Both sensor configurations will be hereinafter named TR 73 Endo 10 and TR 73 Endo 20.

## 4. Experiment 1: Systematic Comparison of the Measuring Volumes by Scanning with a Calibrated Sphere

### 4.1. Experimental Setup

In order to evaluate the appropriateness of the respective sensors for a possible measuring and inspection task, systematic experiments are to be carried out on a calibrated reference object within the course of a comprehensive test series. The calibrated reference object is a optically cooperative ceramic sphere from Kolb & Baumann GmbH & Co. KG (Aschaffenburg, Germany).

**Figure 5:**
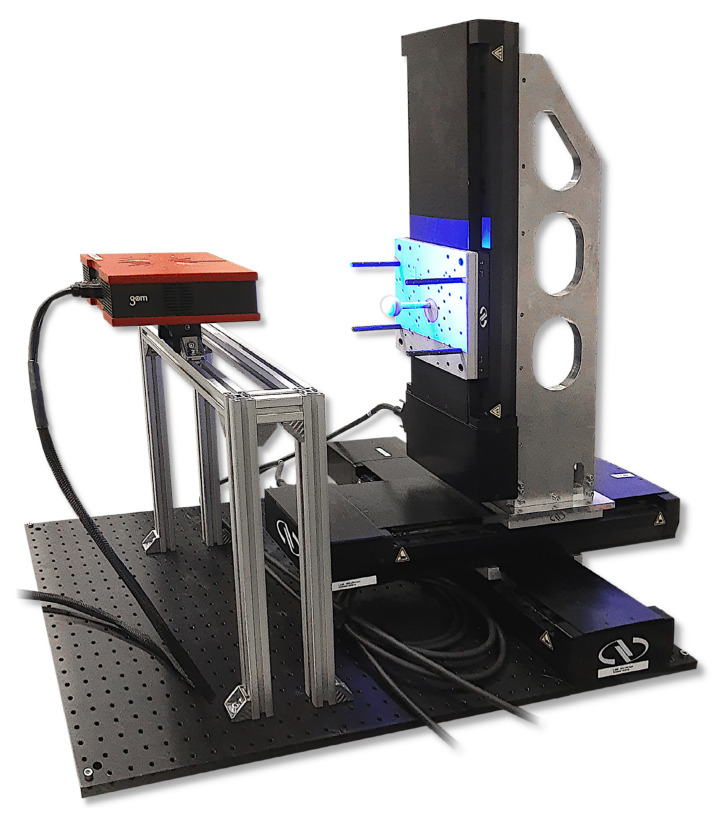
Three-axis experimental set-up for scanning the measuring volume with a calibrated sphere.

Table 2 shows the most important parameters from the calibration certificate of the sphere. In order to be able to evaluate the measuring volume as accurately as possible, the sphere is automatically positioned in front of the respective measuring system. For most of the experiments, a positioning setup as shown in Figure 5 is used. The positioning system features three motorized linear stages of type M-IMS300BPP from Newport Corporation (Irvine, CA, United States) each with a guaranteed positioning accuracy of ±5 µm. Since the maximum travel distance of 300 mm is in the limit of the possible measuring volume of the ATOS Compact Scan 2M, a tactile portal coordinate measuring machine type UPMC 1200 CARAT S-ACC from ZEISS Industrial Metrology (Oberkochen, Germany) is additionally used for sphere positioning. The maximum permissible length measurement deviation (according to DIN EN ISO 10360-2 [49]) is 3.9 µm (E1) at full travel. The grid is set individually for each measuring system, resulting in about 5000 positions. A combination of a fine grid (according to the manufacturer’s specifications) is selected and a larger grid is added to the outer areas of the measuring volume. The distance between the sample points is increased along the optical axis according to the respective angle of view in the field of view. Wherever possible, High dynamic range (HDR) sequences were used to perform the triangulation under optimal exposure settings.

### 4.2. Data Processing

**Figure 6:**
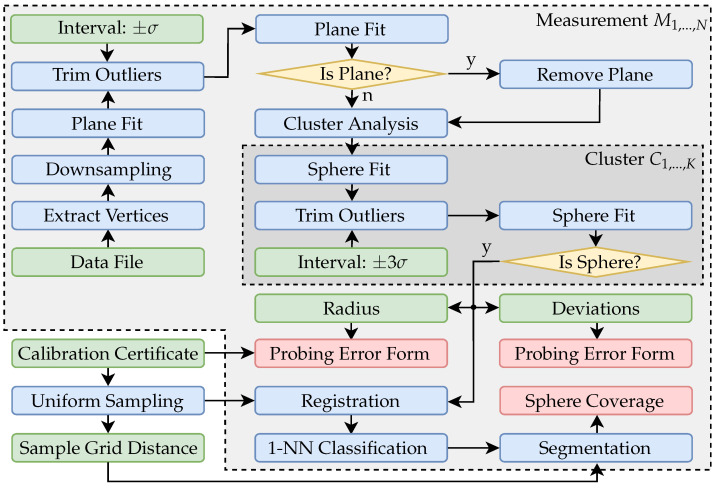
Flowchart with all major data processing operations for each individual measurement.

The positioning and the measuring procedure were automated, and the polygonal *.STL* format was chosen as data interface for the commercial systems. The files are exported with maximum quality. The measurements were carried out in air-conditioned laboratory rooms (ϑ≈ 20 °C) and required several days for each measuring system. The flowchart in Figure 6 shows all major data processing operations, which are necessary in order to calculate the corresponding characteristic parameters for each sphere position. First, the points are extracted from the measurement data. Since the commercial systems already use extensive internal preprocessing and smoothing algorithms, unfortunately, it is not possible to access the actual triangulated data. But, in the application context, it is possible to consider each of these systems as a black box. Therefore, the vertices are extracted from the premeshed *.STL* exchange files. The also applied endoscopic triangulation system from the TCRC 73 provides the pipeline with the actual triangulated point clouds. These were adaptively masked according to signal and calibration characteristics within the typical measurement routines [50]. Random downsampling to 50,000 data points is performed to speed up subsequent computations and to make any registration algorithms converge faster and more robust.

**Figure 7:**
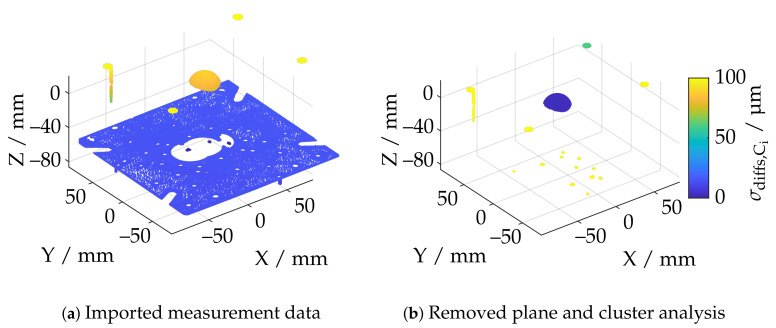
Masking of the base plate and cluster analysis using the example of the ATOSCore 200 5M.

If the point cloud already consists of less data points, no downsampling is performed. Figure 7a shows an exemplary single measurement from the ATOS Core 200 5M. Due to the comparatively large measuring volume, essential components of the experimental setup have also been reconstructed in addition to the actual sphere. This concerns especially the base plate of the vertical axis of the positioning system on which the sphere was mounted. In the first step of the data processing, therefore, it is necessary to remove a possible plane. For this purpose, it is first necessary to determine whether a plane is present in the measurement data at all. Using a common total least-squares approach, a plane was fitted to the data. Since even in the presence of that plane essential parts of the measurement data are not planar, a confidence interval of ±σ was chosen, so that about 31.7 percent of the data with a point distance too large in the plane normal direction were discarded. The planefit was then performed again. All points with a normal distance of less than 10 mm were then assigned to the plane. The evaluation of a plane is based on the standard deviation of all points assigned. The maximum standard deviation has been set to 1 mm, which has proven experimentally to be a robust threshold, since this value is much higher for non-planar datasets, while planar data shows considerably less noise surrounding the fitted planes.

Figure 7b shows that, in the case of a measuring system with a large measuring volume, the remaining point cloud contains further geometric artifacts which are not part of the actual sphere. Based on the *DBSCAN* algorithm [51], this fragmented point cloud is divided into *K* corresponding clusters. Each cluster is then checked individually to determine whether it represents a sphere. For this purpose, a double Sphere-Fit with an inlier interval of ±3σ is carried out in accordance with the specifications of VDI/VDE guideline 2634-2 [28] using a Least-Squares approach. The segmentation of a possible sphere is performed analogous to the procedure for the plane removal via the standard deviation of all deviations of the respective cluster in normal direction. This is illustrated in color in Figure 7b and demonstrates exemplarily that segmentation is feasible. If no sphere is found, the respective measurement will become invalid.

### 4.3. Quality Metric: Probing Error

**Figure 8:**
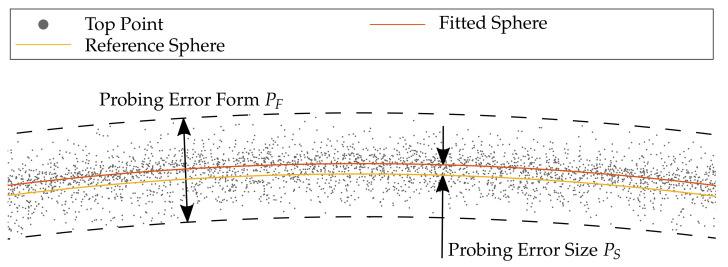
The probing error with respect to form and size of the reconstructed point cloud.

To quantify the quality of each reconstructed sphere within the measuring volume, the metrics of the probing error are applied according to VDI/VDE guideline 2634-2 [28]. As shown in Figure 8, it is required to differentiate between the probing error with respect to form and size. Thus, the probing error according to form $P_{F}$ represents the span of all deviations in normal direction after the numerical fitting of a sphere into the reconstructed point cloud. The span in the following refers to a confidence interval of ±3σ. It should be noted that, in DIN EN ISO 10360-8 [31], the span is defined by 95% of the measuring points regarding probing positions within the cone angle. The probing deviation regarding size $P_{S}$ is the deviation of the reconstructed feature (diameter) from the calibrated diameter according to Table 2.

### 4.4. Quality Metric: Sphere Coverage

Since noise is particularly noticeable in the peripheral areas with a low concentration of points, additional metrics are required to quantify the measurement volume precisely. Otherwise, both very small and high deviation values could obstruct a robust interpretation of the measurement volumes.

**Figure 9:**
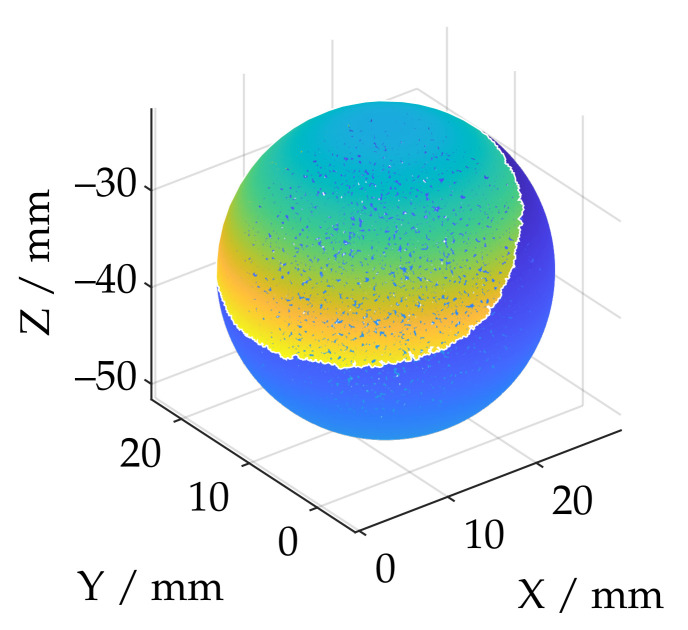
Reconstructed point cloud (ATOS Core 200 5M) with respect to the whole sphere.

**Figure 10:**
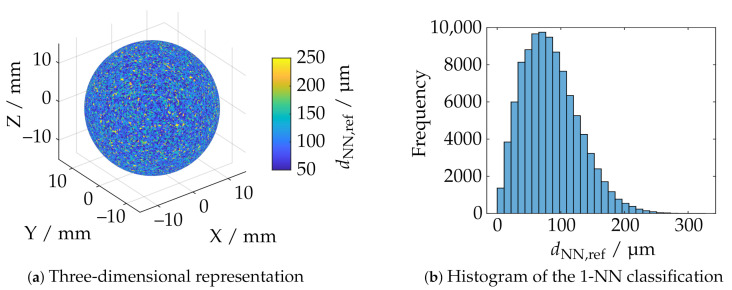
Reference sphere with 100,000 samples and Euclidean distance to the nearest neighbor.

Furthermore, it is also of practical relevance to evaluate the measurement volume with respect to the maximum size of the geometry to be reconstructed. In order to quantify the size of the measuring volume in the terms of the triangulation basis through overlapping of the camera and projector cones of view, which are limited by the depth of field, an additional metric is introduced as a supplement to the deviation-based evaluation: the sphere coverage. According to Figure 9, this specifies the proportional coverage of the actual sphere after the reconstruction. To estimate the sphere coverage, a reference point cloud is sampled using the Cook [52] and Marsaglia [53] point picking approach. This provides a uniform distribution on the surface of a unit sphere, which is then scaled by the actual diameter from the calibration certificate. Figure 10a shows a reference point cloud $P_{\mathrm{ref}}=\left\{p_{\mathrm{ref},1},...,p_{\mathrm{ref},n}\right\}$ with n= 100,000 sample points $P_{\mathrm{ref}}\in R^{3}$. The Iterative closest point (ICP) algorithm [54,55] is then used to register the reconstructed sphere segment from the current measurement $P_{M}=\left\{p_{M,1},...,p_{M,m}\right\},\left|P_{M}\right|=m$ to the reference point cloud $P_{\mathrm{ref}}$, where *M* is the number of nearest neighbors (NN). The corresponding distances of the set of nearest neighbors ${}^{M}N_{\mathrm{ref}}={\{}^{M}n_{\mathrm{ref},1},...{,}^{M}n_{\mathrm{ref},n}\}$ from $P_{\mathrm{ref}}$ to $P_{M}$ are determined by a 1-NN classification using the fast *k*-d-search tree [56]. Let *D*: $R^{3\times m}\times R^{3\times n}\to N_{0}^{+}$ be a linear function, which represents the sum of all unique points:

(1) $$V_{\mathrm{ref},M}=\{n_{1},...,n_{k}\;|\;∥n_{i}-p_{i}{∥}_{2}<d_{\mathrm{NN}}\;\forall \;i=1,...,k,\;\;p_{i}\in P_{\mathrm{ref}},\;n_{i}\in N_{\mathrm{ref}},\;k\leq n\}$$

of the respective classification in accordance with the neighborhood limitation by the threshold value $d_{\mathrm{NN}}$ with $\cap_{i=1}^{k}n_{i}=\emptyset$. The absolute sphere coverage

(2) $$A_{\mathrm{abs}}=\frac{D(P_{\mathrm{ref}},P_{M})}{n}=\frac{|V_{\mathrm{ref},M}|}{n}$$

is the quotient of the sum of unique neighbor points and the total reference samples.

The relative sphere coverage $A_{\mathrm{rel}}$ is obtained by normalizing $A_{\mathrm{abs}}$ to the maximum that applies to all measuring poses. The determination of $d_{\mathrm{NN}}$ is dependent on the size of the sphere and the number of generated reference samples *n*, which both influence the mean distance to the respective next neighbor $\bar{d_{\mathrm{NN},\mathrm{ref}}}$ within $P_{\mathrm{ref}}$. The histogram in Figure 10b shows the sample grid distance of the reference point cloud for the corresponding sample number and diameter. The threshold $d_{\mathrm{NN}}$ is set to 1.5 $\bar{d_{\mathrm{NN},\mathrm{ref}}}$, i.e., about 125 µm. The determination of $\bar{d_{\mathrm{NN},\mathrm{ref}}}$ is based on a further NN-classification within $P_{\mathrm{ref}}$ where the nearest neighbor was searched, which is not the same as the initial point. It has been observed experimentally that it is possible to achieve good comparability from a masking of the dataset at $0.5\leq A_{\mathrm{rel}}\leq 0.9$ and, thus, remove sensor positions if the total reconstruction is too small.

In order to relate the introduced metrics of the sphere coverage more generically to the context of the actual investigation of the maximum possible reconstructable surface, it is possible to calculate the total area from the proportional absolute coverage, where

(3) $$A_{\mathrm{total}}=d^{2}\pi A_{\mathrm{abs}}$$

represents the total area of a reconstructed pose. It is, however, to be noted that this information always refers to a sphere with a fixed diameter according to Table 2. In addition, many measuring systems do not require the entire measuring range (MR) to reconstruct the sphere for each measuring pose. This suggests that $A_{\mathrm{total}}$ should not be considered equal to the maximum reconstructable surface area of each measuring pose. Nevertheless, there is a good proportionality between the maximum possible surface reconstruction and the relative sphere size $A_{\mathrm{ref}}$, which is, therefore, used in the following as the main reference for limiting the measurement volume in terms of reconstructable surface area.

### 4.5. Outlier Removal

**Figure 11:**
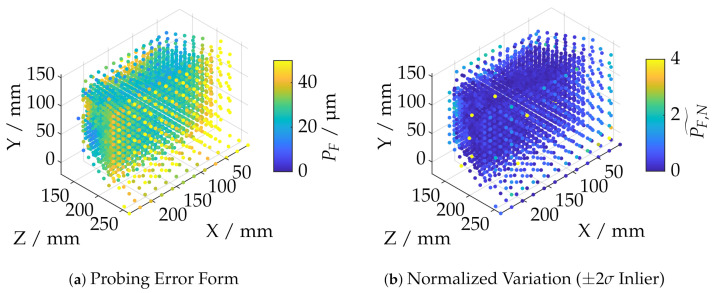
Visualization of the outlier identification on the example of the dataset of the ATOS Core 200 5M at $A_{\mathrm{rel}}\geq 0.5$.

The ISO/IEC guideline 98-3:2008 [57] (also referred to as JCGM 100) recommends 20 repeated measurements for each sensor pose in order to compensate for stochastic influences on the measuring result. This is not feasible due to the high number of measuring poses (*n* ≈ 5.000) per measuring system with regard to absolute experiment duration and generated data volumes. In order to correct the data of implausible outlier measurements, additional outlier masking is performed. Let the probing error $P_{F}$:$R^{3}\to R^{1}$ be a function which maps each sampled reference point $p_{i}\in P_{s}=\left\{p_{1},...,p_{n}\right\}$ to a corresponding probing error value. A subset $P_{\mathrm{NN},j}\subset P_{s}=\left\{p_{\mathrm{NN},j,i},...,p_{\mathrm{NN},j,m}\right\}$ with m≤n of all points $P_{s}$ for each sample point $p_{i}$

(4) $$P_{\mathrm{NN},j}=\{p_{j}\;|\;∥p_{i}-p_{j}{∥}_{2}<d_{c}\;\forall \;\underset{i\neq j}{p_{i}}\in P_{s},\;\;\;|P_{\mathrm{NN}}\left|=m,\;\right|P_{s}\left|=n\right\}$$

results by applying a rangesearch operation with cut-off distance $d_{c}$. This corresponds to a *K*-NN classification which is also based on the *k*-d-search tree. Therefore, *K* is adjusted for each sample point $p_{i}$ so that all neighboring points of subset $P_{\mathrm{NN},j}$ lie within a sphere. The cut-off distance $d_{c}$ is set to ≈10 $d_{\mathrm{grid}}$, where $d_{\mathrm{grid}}$ represents the average grid distance in the high-resolution area of the sample point cloud. Figure 11a shows an example of the probing error with each corresponding sample position in which relative sphere coverage $A_{\mathrm{rel}}$ according to Section 6.4 is at least 50%. The average grid distance $d_{\mathrm{grid}}$ is determined by a further 1-NN classification.

**Figure 12:**
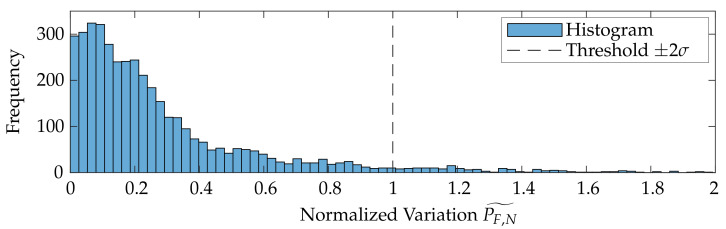
Histogram of the normalized variation values $\tilde{P_{F,N}}$.

The corresponding variation metric $\tilde{P_{F}}$,

(5) $$\tilde{P_{F}}\left(p_{i}\right)=\left|P_{F}\left(p_{i}\right)-median\left(P_{F}\left(P_{\mathrm{NN},j}\right)\right)\right|,$$

to trim possible outliers is given by the difference of the probing error $P_{F}$ with respect to sample point $p_{i}$ and the median of the probing errors of the respective neighborhood subset $P_{\mathrm{NN},j}$ as shown in Figure 11b. $\tilde{P_{F,N}}$ is the variation metric $P_{F}$ normalized to the confidence interval of ±2σ. This interval was found experimentally for all datasets. Possible outlier values are recognizable and rejected according to the histogram in Figure 12.

### 4.6. Uncertainty Considerations

According to VDI/VDE guideline 2634-2 [28], the uncertainty of the respective probing error

(6) $$u\left(P\right)=\sqrt{{\left(\frac{F}{2}\right)}^{2}+u^{2}\left(F\right)}$$

is influenced by the form deviation of the sphere *F* and the uncertainty of the form deviation u(F). Due to the measurement setup under laboratory conditions with optimal exposure settings, further factors contributing to the uncertainty can be neglected. If the calibration certificate states the expanded calibration uncertainty U(F) of the form deviation, then the standard uncertainty,

(7) $$u\left(F\right)=\frac{U(F)}{k},$$

is calculated by the coverage factor *k* (usually k=2). According to Table 2, the uncertainty u(P) in this case is 0.43 µm. Due to the single measurement of the sphere at each position, the application of VDI/VDE 2634-3 [27] for multiple views is not necessary.

The uncertainties of the commercial 3D scanners of GOM GmbH (Table 1) were determined by the manufacturers using VDI/VDE 2634-3 [27] in combination with DIN EN ISO 14253-1 [29]. Due to the available uncertainty data and the large number of measurement positions, a separate measurement uncertainty analysis can be dispensed with. Weickmann [58] examined the probing uncertainty individually for each point depending on the relative surface inclination. The results show a deterioration of the uncertainty with increasing inclination angle, but no correlation of the uncertainties with the measurement positions.

## 5. Experiment 2: Effects of the Different Measurement Systems on the Reconstruction of Process Related Geometric Features

**Figure 13:**
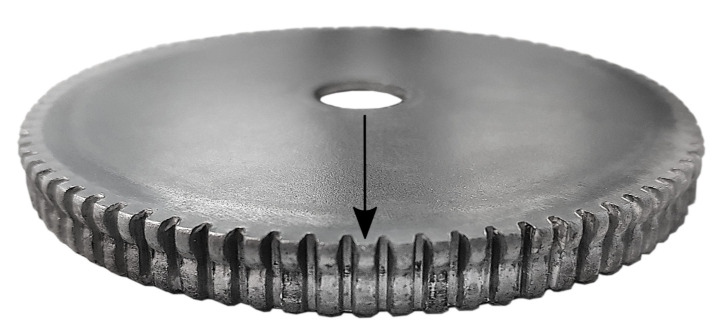
Specimen part (process component 2) with the examined tooth (marked with arrow).

**Figure 14:**
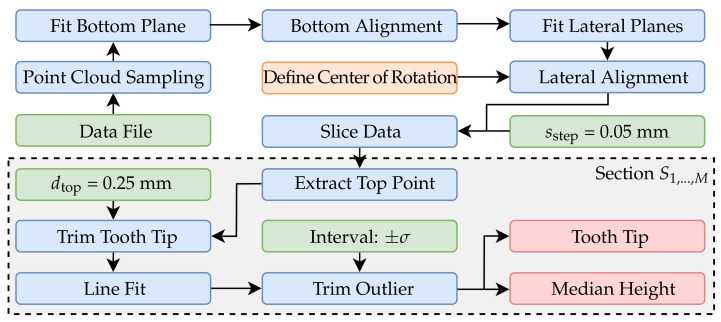
Flowchart to align the reconstructed part of a SBMF gear in three dimensions and to derive the tooth height.

In a second experiment, geometric features of a process-related specimen will be reconstructed and compared in order to supplement the investigations of the measurement volumes from Section 4 with practical applications and to relate the observations to the introduced metrological problems. In this case, a tooth of a SBMF workpiece is reconstructed and evaluated with respect to tooth height and overall upper surface. Figure 13 shows the examined specimen part, which corresponds to process component 2 from Figure 2b. According to the flowchart in Figure 14, an alternative approach is used in which the differently sized data are aligned in three dimensions to derive the reconstructed tooth height. First, a point cloud with uniform point density is generated from the polygonal measurement data using the approach Osada et al. [59].

**Figure 15:**
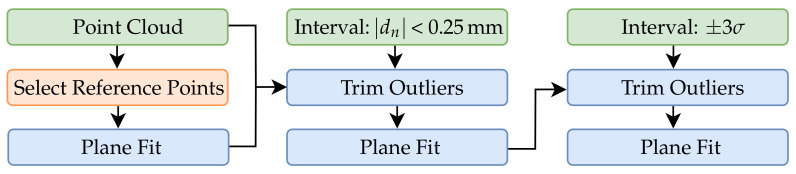
Flowchart for plane fitting with initial manual assignment.

**Figure 16:**
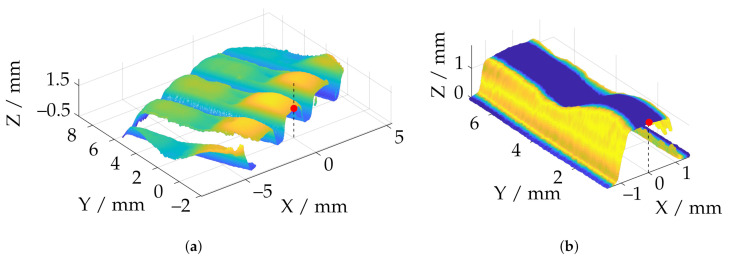
Overview of the lateral alignment of a reconstruction with the TR 73 Endo 20 endoscope (red: center of rotation; dashed line: axis of rotation). (**a**) Point cloud registered to the tooth base plane with manually selected rotation center; (**b**) Aligned and trimmed point cloud after optimization with respect to the lateral surfaces.

In the subsequent step, the tooth base is aligned. Approximately, in smaller sections, this can be considered as a plane. The plane is first prealigned via a subset of six manually assigned points by estimating an optimal rotation and translation using partial fraction decomposition via the approach of Kabsch et al. [60]. In the next iteration, a subset of all points in which deviation in normal direction $d_{n}$ to the manually assigned plane is smaller than 0.25 mm are applied for a two-step total least squares fitting according to Section 4 with ±3σ inlier. The algorithm for plane fitting with manual assignment is shown in Figure 15. For the following registration of the reconstructed data after the base plane has been defined, the lateral surfaces are extracted from the data (Figure 16b). For this purpose, the algorithm described in Figure 15 is applied separately for each lateral surface.

For the rotation $\theta_{z}$ around the Z-axis (dashed line in Figure 16), a center of rotation is manually assigned from the dataset. Translational shifts can be corrected and optimized later (see Section 7). For the subsequent lateral alignment, the deviation of the fitted planes from an ideal symmetrical alignment with respect to the YZ plane is then minimized. The minimization is based on the widely used nonlinear solver through the Levenberg-Marquardt algorithm [61,62]. Figure 16b shows a final aligned and trimmed example measurement. It can also be observed that the height profile of the tooth derived from the geometry is very uneven along the Y-axis. Due to the different forming forces and the demonstrator characteristics of the formed workpiece, uniform height profiles over the entire tooth are not available. Nevertheless, the tooth height is an important parameter for characterizing the forming process.

**Figure 17:**
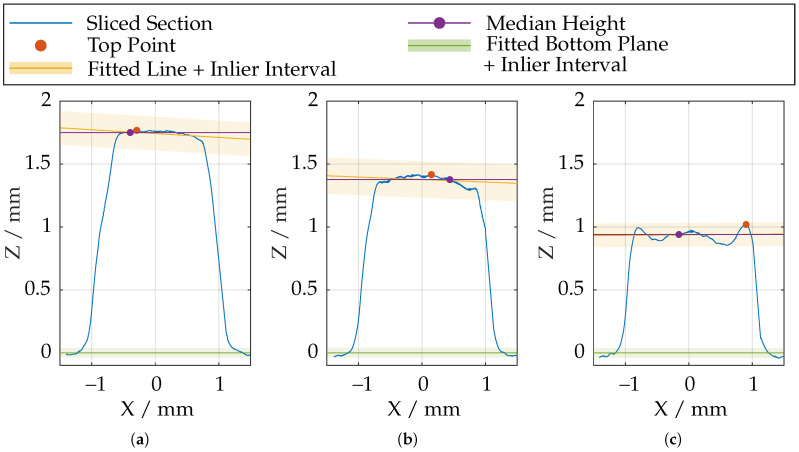
Height profile of the examined gear tooth at various slicing positions along the tooth. (**a**) Highest elevation; (**b**) Medium elevation; (**c**) Lowest elevation.

Therefore, the geometry is divided into different sections, with distance $s_{\mathrm{step}}$ = 0.05 mm and evaluated separately. According to Figure 14, the point with the largest Z-coordinate is selected, and a line is fitted into the data points with maximum distance $d_{\mathrm{top}}$ = 0.25 mm. The fitted lines are shown in Figure 17 for different slice positions. To obtain a unambiguous height coordinate, the *median* is extracted from the data points in a ±σ inlier interval of the fitted line. The points in this interval are also used to subsequently reassemble the entire geometry of the tooth tip from the individual sections.

An evaluation of the characteristic values according to DIN ISO 1328-1 [63] is not possible due to the partially incomplete teeth reconstruction at different point densities.

## 6. Results—Experiment 1: Systematic Comparison of the Measuring Volumes by Scanning with a Calibrated Sphere

### 6.1. Sphere Coverage $A_{\mathrm{abs}}$

**Figure 18:**
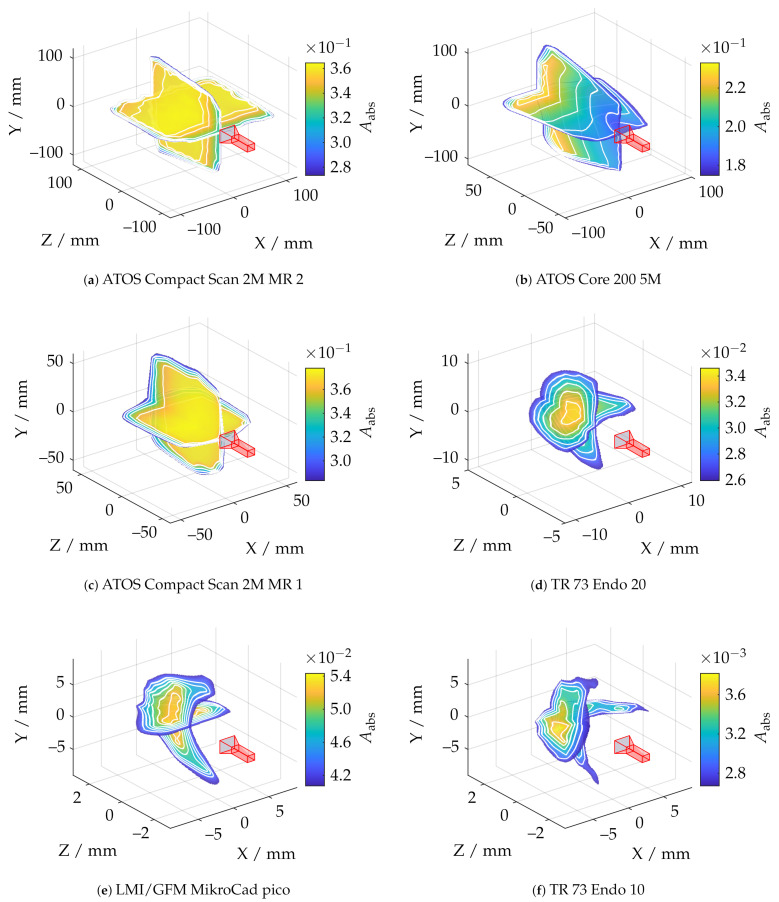
Comparison of all applied measuring systems with respect to the absolute sphere coverage $A_{\mathrm{abs}}$ and pre-masking according to $A_{\mathrm{rel}}\geq 0.5$.

**Figure 19:**
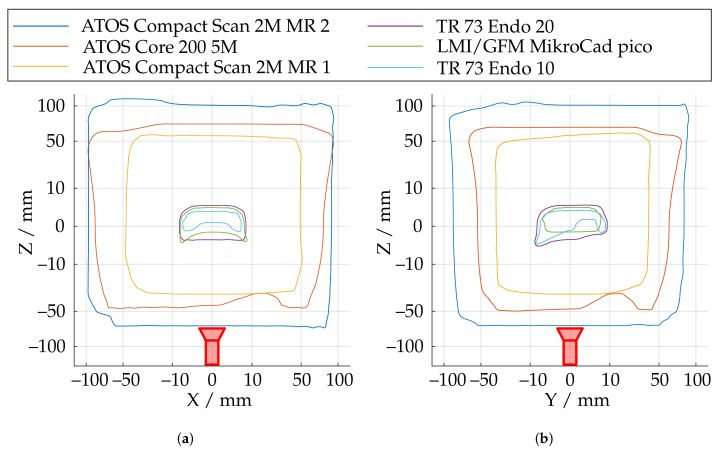
Shape of the measurement range at a fixed threshold of $A_{\mathrm{rel}}\geq 0.8$ at two perpendicular slicing positions. (**a**) Cross section in the plane of the triangulation base. (**b**) Cross section perpendicular to the triangulation base..

Figure 18 illustrates the measurement ranges with respect to the absolute spherical coverage $A_{\mathrm{abs}}$. The sensors, shown in red, were aligned so that the triangulation base is located in the ZX plane. $A_{\mathrm{abs}}$ represents the best possible usability of the measurement volume with respect to maximum reconstructable geometry. The scale effect can be observed well, since $A_{\mathrm{abs}}$ varies from about 0.38% to 38%. It can also be observed that the ATOS Core 200 5M has a slightly more non-uniform measuring volume than the ATOS Compact Scan 2M. In the area of the smaller scales, all sensors show a more significant influence of field curvature, which results in curving of the measuring range to a spherical segment in Z direction. This effect is most significant with the LMI/GFM MikroCad pico sensor and should always be taken into account when positioning the instrument. On the other hand, the maximum sphere coverage of about 5.4% is the highest in this scale range. The endoscope with 10 mm working distance (TR73 Endo 10) shows a comparatively inhomogeneous shape of the measuring volume. This may be due to the optics used with a comparatively low depth of field which also results in a significantly reduced sphere coverage. The TR73 Endo 20 sensor has a more uniform appearance of the measuring volume with the same triangulation angle and and a much higher maximum sphere coverage. Figure 19 shows the shape of the measuring range according to cross sections from Figure 18 for a fixed threshold value of $A_{\mathrm{rel}}\geq 0.8$. Thus, it can be observed that the measuring range in the plane of the triangulation base is considerably more symmetrical, larger, and more uniform. Perpendicular to this, especially the endoscopic sensors show a certain directional orientation.

### 6.2. Probing Error Size $P_{S}$

**Figure 20:**
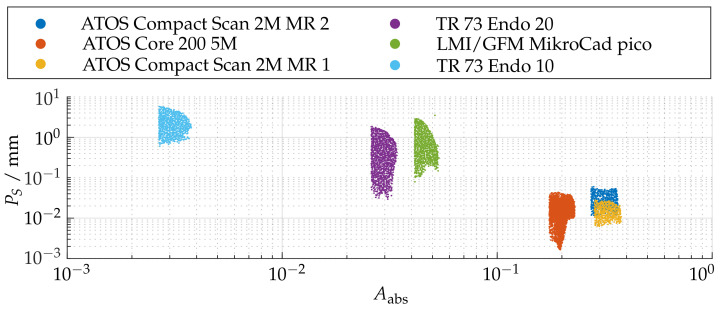
Relation between the absolute sphere coverage $A_{\mathrm{abs}}$ and the probing error $P_{S}$ after pre-masking according to $A_{\mathrm{rel}}\geq 0.5$.

Figure 20 shows for each measuring pose the relation between the sphere coverage $A_{\mathrm{abs}}$ and the probing error $P_{S}$. It can be observed that, if a reconstruction of less than 10% of the total sphere is performed, the errors increase significantly.

However, this is to be anticipated, since the reconstruction of the radius feature is increasingly affected by uncertainties for smaller partial reconstructions of the sphere. Therefore, the comparison of the probing error $P_{S}$ is only carried out for the three sensors with $A_{\mathrm{abs}}\geq 0.1$. It can be observed that the corresponding probing deviation scales with the size of the measuring range. Similarly, all sensors show the best possible results in the foreground area of the measuring range. The ATOS Core 200 5M shows additionally an diagonal bias.

**Figure 21:**
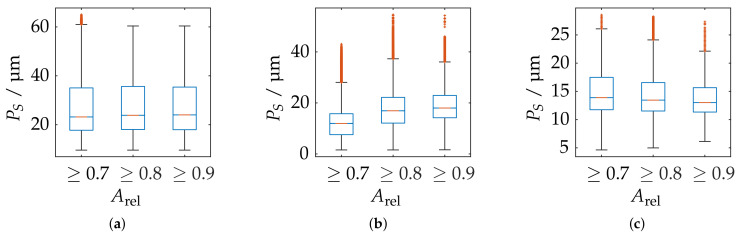
Statistical representation of the probing error form $P_{F}$ of all measuring poses with limitation of the measuring range by different threshold values of the relative spherical coverage $A_{\mathrm{rel}}$. Orange line: Median, Blue box: Upper and lower quartile, Whisker = 1.5 (99.3 percent coverage), Orange dots: Outlier. (**a**) ATOS Compact Scan 2M MR 2. (**b**) ATOS Core 200 5M. (**c**) ATOS Compact Scan 2M MR 1.

**Figure 22:**
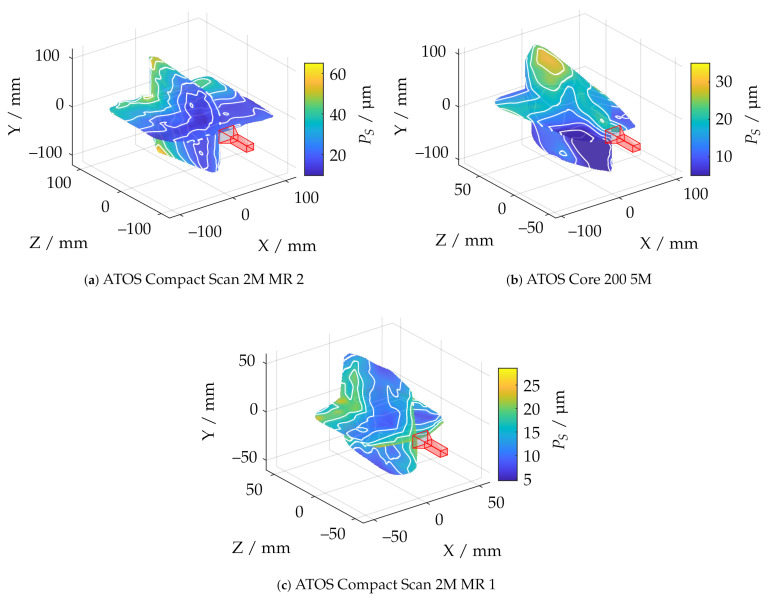
Comparison of all measuring systems with $A_{\mathrm{abs}}\geq 0.1$ and in correlation to the probing error size $P_{S}$ and pre-masking according to $A_{\mathrm{rel}}\geq 0.5$.

In order to further correlate the probing deviation with the actual measurement volume according to the greatest possible reconstruction, Figure 21 shows a static evaluation for different measurement volumes according to $A_{\mathrm{rel}}$ for all measurement poses. It is noticeable that, in the area of the largest possible reconstruction, the best results are not necessarily reconstructed according to $P_{F}$. This is only the case for the ATOS Compact Scan 2M sensor at MR 1. This impression is also confirmed when comparing Figure 18 and Figure 22. Therefore, when selecting and positioning the sensors, thus, it is required to consider between maximum reconstruction and minimum probing error size $P_{S}$.

### 6.3. Probing Error Form $P_{F}$

**Figure 23:**
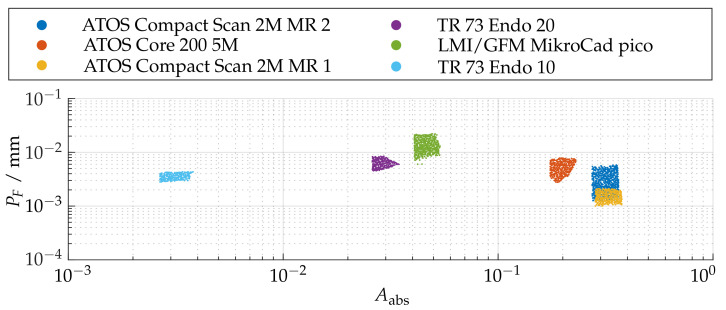
Relation between the absolute sphere coverage $A_{\mathrm{abs}}$ and the probing error $P_{F}$ after pre-masking according to $A_{\mathrm{rel}}\geq 0.5$.

Figure 23 shows the probing error of form $P_{F}$ with respect to absolute sphere coverage $A_{\mathrm{abs}}$ for all sensor poses. It is apparent that the probing errors for each measurement system are concentrated in comparable ranges. However, it can also be observed that the sensors with smaller measuring ranges and lower working distances perform comparatively poorly in this experiment. This is particularly noticeable with the LMI/GFM MikroCad pico sensor, although it already provides postprocessed mesh data. Therefore, it was also investigated whether this is caused by only extracting the vertices from the geometric data by creating additional random samples in each polygon using the approach of Osada et al. [59]. However, this had no quantifiable influence on the results.

**Figure 24:**
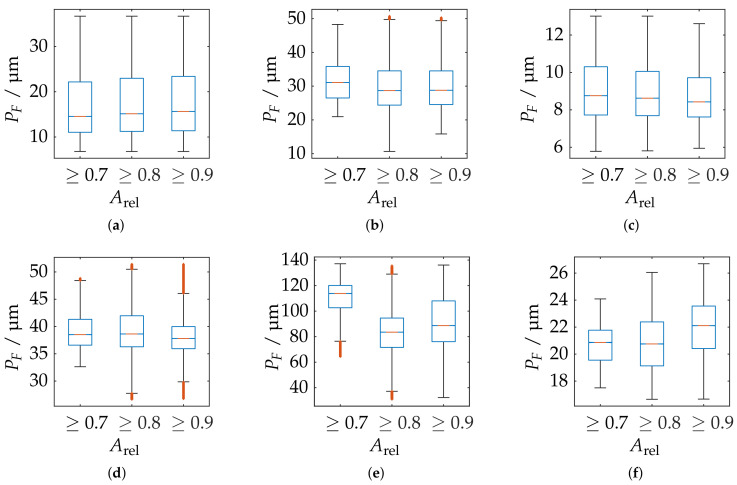
Statistical representation of the probing error form $P_{F}$ of all measuring poses with limitation of the measuring range by different threshold values of the relative spherical coverage $A_{\mathrm{rel}}$. Orange line: Median, Blue box: Upper and lower quartile, Whisker = 1.5 (99.3 percent coverage), Orange dots: Outlier. (**a**) ATOS Compact Scan 2M MR 2. (**b**) ATOS Core 200 5M. (**c**) ATOS Compact Scan 2M MR 1. (**d**) TR 73 Endo 20. (**e**) LMI/GFM MikroCad pico. (**f**) TR 73 Endo 10.

**Figure 25:**
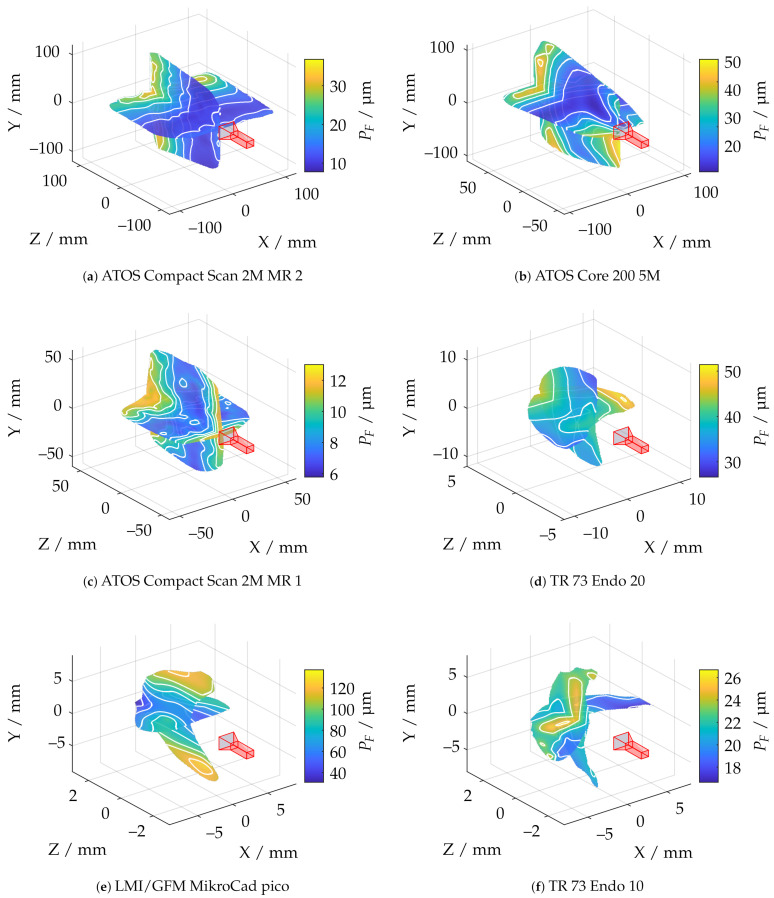
Comparison of all applied measuring systems with respect to the probing error form $P_{F}$ and pre-masking according to $A_{\mathrm{rel}}\geq 0.5$.

As shown in Figure 24e, the LMI/GFM MikroCad pico sensor exhibits a significant reduction of the probing error form $P_{F}$ when masking the measurement volume around areas with larger surface reconstruction according to $A_{\mathrm{rel}}$. However, a significant noise increase can be observed in the YZ plane, perpendicular to the triangulation base (see Figure 25. According to Figure 25f, the TR73 Endo 10 sensor at 10 mm working distance shows overall good results due to its higher magnification, which is presumably lead to an even, low-noise reconstructed surface. Following Figure 24f, a trade-off can be observed in comparison to the maximum reconstructed surface according to $A_{\mathrm{rel}}$. Both of the two endoscopic sensors suggest that the best results cannot be achieved in the center of the measuring range. The other sensors show a similar behavior as for the probing error with respect to size ($P_{S}$). However, as shown in Figure 25b, it is noticeable that the ATOS Core 200 5M sensor only has an influence in the Z-direction and its probing error form $P_{F}$ is exceeded compared to both measuring ranges of the ATOS Compact Scan 2M.

### 6.4. Size of the Measuring Volume

**Figure 26:**
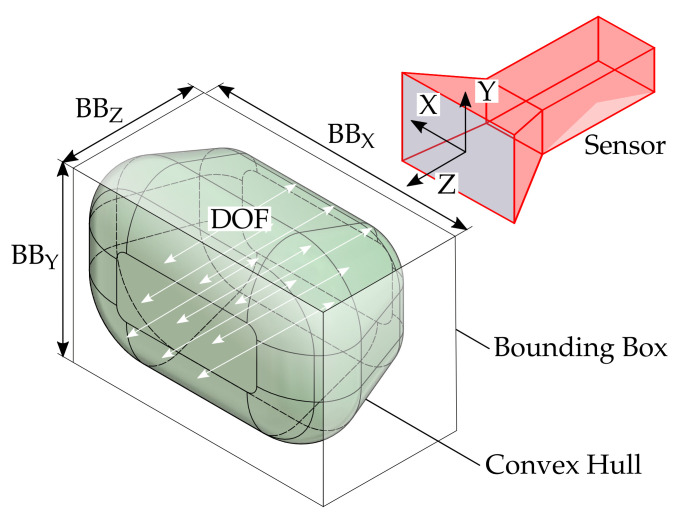
Visualization of a measuring volume and possible geometrical characteristics.

**Figure 27:**
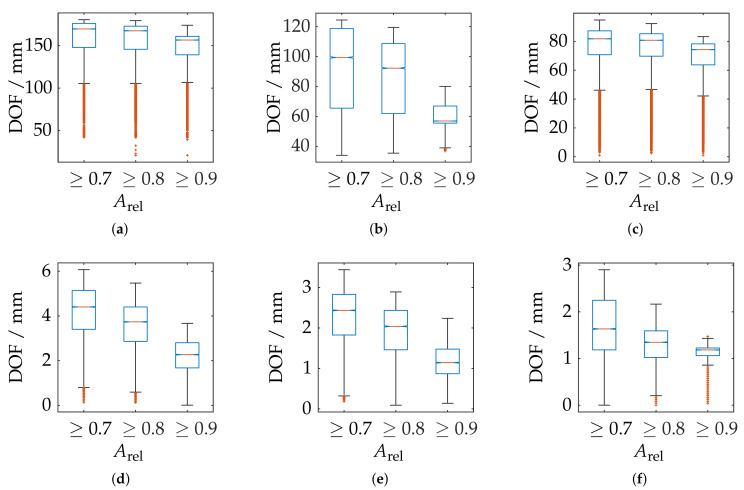
Statistical evaluation of the depth of field (DOF) of all measuring poses with limitation of the measuring range by different threshold values of the relative spherical coverage $A_{\mathrm{rel}}$. Orange line: Median, Blue box: Upper and lower quartile, Whisker = 1.5 (99.3 percent coverage), Orange dots: Outlier. (**a**) ATOS Compact Scan 2M MR 2. (**b**) ATOS Core 200 5M. (**c**) ATOS Compact Scan 2M MR 1. (**d**) TR 73 Endo 20. (**e**) LMI/GFM MikroCad pico. (**f**) TR 73 Endo 10.

As a supplement to the quantitative assessment via the maximum reconstructed surface area, the measurement ranges shall be further quantified, which is shown in an exemplary shape in Figure 26. On the one hand, it is possible to specify the dimensions of the bounding box. This is a very practical approach, but it does not provide any information about the actual shape of the measuring volume. Therefore, the volume of the convex hull is calculated additionally. The depth of field (DOF) is the span in the direction of the optical axis for each set of sample points with identical X and Y components within the convex hull. Since this parameter is also strongly dependent on the position in the measuring volume, additional tolerances will be specified according to Figure 27. The further results are listed in Table 3.

It can be observed and quantitatively demonstrated that the measuring volumes of both measuring ranges of the ATOS Compact Scan 2M are very uniform and constant. All other sensors show a strong influence of the increasing masking according to $A_{\mathrm{rel}}$. The total volume of the convex hull varies by several orders of magnitude, underlining the different scale ranges of all sensors. The depth of field of the 10 mm endoscope (TR73 Endo 10) has been assumed to be about 2 mm. This is generally only apparent with a relative sphere coverage $A_{\mathrm{rel}}$ of 70 percent. A reduction of the maximum possible surface area seem to be necessary in order to use this sensor effectively.

## 7. Results—Experiment 2: Systematic Comparison of the Measuring Volumes by Scanning with a Calibrated Sphere

Before discussing the results, it has to be mentioned that measurements on the sample parts are rendered partially problematic due to the reflectivity properties of the corresponding technical surface. The degree of reflectivity also depends, in particular, on the arrangement or sensor pose. Due to the high forming forces in the area of the formed teeth, very low roughness is achieved in these areas, which leads to more specular reflectivity and renders measurements by means of active triangulation difficult. Nevertheless, this method represents a good compromise in SBMF forming for real measurements by reconstructing thousands of object points within seconds or less. Therefore, the following results are intended to demonstrate the suitability of the sensors in a practical context only and are not intended to represent the full potential accuracy.

In order to put the results in a better context and to provide a ground truth for the reconstructed data, a comparison with measurements of a confocal laser scanning microscope (CLSM) of the type VK-X 210 from the manufacturer Keyence Corporation (Osaka, Japan), at an objective with 10× magnification and a lateral resolution of approximately 160 nm, is provided. Since the tooth flanks cannot be robustly reconstructed due to the corresponding aperture angles, these datasets were removed and only the tooth tip and root were considered. Furthermore, due to the small measurement range, stitching had to be performed.

**Figure 28:**
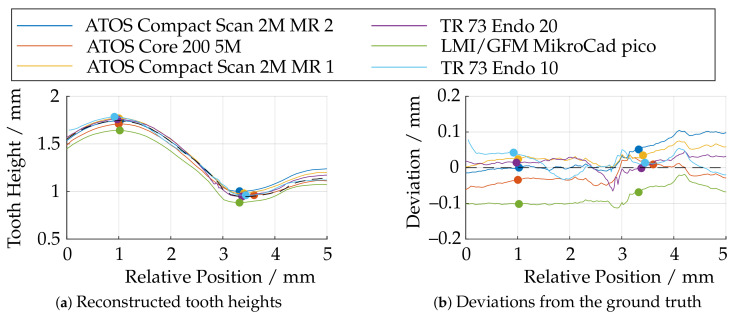
Comparison of the reconstructed tooth heights as measured on the specimen and deviations with respect to a confocal laser scanning microscope (CLSM) measurement.

Figure 28a shows the tooth heights derived from the reconstructed datasets using the algorithms in Section 5. The black dashed line here indicates the reference data from the CLSM. In order to compare the data in the best possible way, the measurement data were shifted in such a way that the sum of the squared deviations of the individual measurements from the reference measurement data was minimized via a nonlinear optimization. The remaining residual deviations from the reference data are shown in Figure 28b. The minimum and maximum tooth heights were additionally marked.

Basically, the LMI/GFM MikroCad pico sensor performs poorest in this experiment, as well, since the tooth height is reconstructed too small over the entire tooth length. The sensors of the type ATOS Core 200 5M and ATOS Compact Scan 2M show partially good results, but have certain deviations over the entire tooth length either in the front or in the back part. The 3D endoscope at 20 mm working distance (TR73 Endo 20) provides good results, showing larger deviations only in the transition area at a relative position of 3 mm. When using the optics with a working distance of 10 mm (TR73 Endo 10), there are significantly larger deviations, although theoretically a more uniform surface reconstruction would have been expected according to Figure 18. Here, the depth of field, which is too small for this experiment (see Figure 27f), appears to be of greater importance.

**Figure 29:**
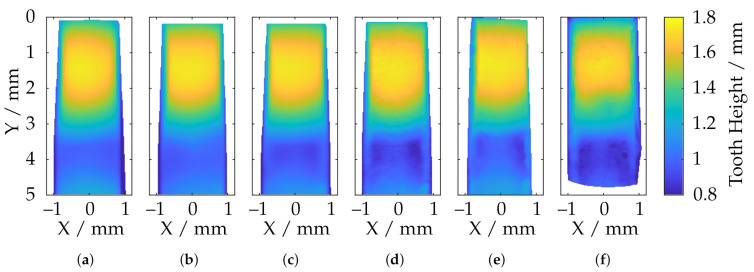
Comparison of the reconstruction of different fringe projection sensors with respect to the height of the tooth tip of the specimen after alignment with reference data (**a**) ATOS Compact Scan 2M MR 2; (**b**) ATOS CORE 200 5M; (**c**) ATOS Compact Scan 2M MR 1; (**d**) TR73 Endo 20; (**e**) LMI/GFM MikroCad pico; (**f**) TR73 Endo 10.

**Figure 30:**
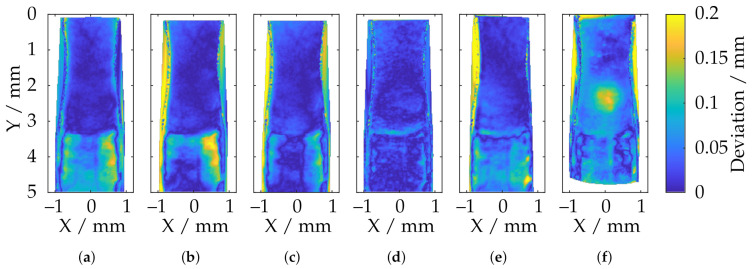
Comparison of the reconstruction of different fringe projection sensors with respect to the deviation of the tooth tip of the specimen with respect to reference data after alignment (**a**) ATOS Compact Scan 2M MR 2; (**b**) ATOS CORE 200 5M; (**c**) ATOS Compact Scan 2M MR 1; (**d**) TR73 Endo 20; (**e**) LMI/GFM MikroCad pico; (**f**) TR73 Endo 10.

For further, detailed investigations of the local reconstruction, only the tooth tips are compared in the following. For this purpose, these areas were extracted from the data and registered to the reference measurements of the CLSM. The (trimmed) ICP algorithm was revealed to be numerically too unstable for this purpose, and since the data had already been rotationally aligned very accurately according to Section 5, only a translational optimization via a least squares approach was performed. The cost function is given by the sum of the squared deviations. An overview is shown in Figure 29. Between the highest and lowest point of the tooth (Y≈ 3 mm), there is some necking of the metal sheet, all of which can no longer be reconstructed by the sensors with a large measuring volume. This may be due to the low lateral and axial resolution (see Table 1). Figure 30 shows additionally the absolute deviations (in Z-direction) from the reference data.

In particular, it can be seen here that, in the area of the necking (3 mm ≤ Y ≤ 5 mm), the measuring systems with a larger measuring volume provide a significantly inaccurate reconstruction due to the lack of resolution. It can be seen that the overall deviations decrease as the measurement volume size decreases and the axial and lateral resolutions increase. The smooth elevation on the front part of the tooth tip (0 mm ≤ Y ≤ 3 mm), on the other hand, is reconstructed well. By far, the best results are achieved by the TR 73 Endo 20 endoscope according to Figure 30d. This is consistent with the observations from Figure 28b, although, here, too, there are smaller deviation spikes in the transition area between the smooth elevation and the necking (Y ≈ 3 mm). It is noteworthy that, according to Figure 30f, in addition to the overall higher deviations, a significant deviation singularity appears in the range of 2 mm ≤ Y ≤ 3 mm for the TR73 Endo 10 endoscope. As already outlined, this sensor should provide the most uniform data and deliver good results, especially in this smooth region near the upper part of the tooth head. This may be due to the limits of the measurement range as described and may be related to poorly calibrated or uncalibrated areas. It is also conceivable that these effects are caused by specular reflections due to the highly reflective technical surface of the gear.

## 8. Discussion

The scalability of fringe projection profilometry in particular enables this measurement technology to be used in a wide range of applications in production metrology and quality assurance. One of the main challenges associated with the application of such technology is the trade-off between the choice of sensor and the correct positioning of the sensor in relation to the specimen. The design of the sensor, in especially the selected imaging optics and the arrangement within the triangulaton base, poses a trade-off between the potential measurement volume and the accuracies that can be achieved. Thus, a wide variety of commercial sensors have positioned themselves on the market in recent years, which are of particular importance for highly innovative manufacturing technologies, such as sheet-bulk metal forming. Due to the wide variety of sub-disciplines and ongoing research within sheet-bulk metal forming, accurate, high-resolution, and holistic three-dimensional reconstruction at different scales is of particular relevance. Measurement time and automatability are additional factors to consider.

The major contribution of this article includes systematic investigations to quantify influencing factors on the local measurement uncertainty and to describe the relative measuring range of different commercial and new developed fringe projection devices across various scale ranges. The probing deviation with respect to form and size has proven to be a suitable metric, whereby it has been observed that the size deviation can be robustly determined only from a total reconstruction of the calibrated reference sphere of at least ten percent. In addition, with the spherical coverage, a further metric was introduced which reflects the maximum reconstruction with respect to the triangulation basis. The advantage of the experiments conducted in this study is that the corresponding metrics are not quantified and located in a general way, but in very high resolution over the entire measuring volume of each sensor. Thus, it is possible to evaluate very precisely for each sensor in which area best results can be expected depending on which metric is applied. It has been shown that the optimal area for all metrics is rarely located at the same position within the measurement volume. The experiments seem to favor in particular the commercial measuring systems in larger scale ranges, since the expected advantage regarding the most uniform surface due to the higher lateral and axial resolution of the instruments with smaller measuring volumes was not observed to the extent it possibly could have been expected. The reasons for this may be manifold and may, for example, be related in particular to the extensive post-processing steps of the commercial systems. However, it should also be mentioned that, due to the spatial frequency of the specimen, no particular advantage can be expected with respect to the relative phase measurement uncertainty with additional magnification. In general, this implies that, for each spatial frequency, there is an optimal corresponding fringe frequency [39,64] of which there is a necessary corresponding pixel resolution. The applied sphere appears to be particularly smoothly reconstructed by certain sensors at larger measuring volumes, suggesting that the spatial frequency of the sphere is favouring sensors with larger scale ranges and aliasing effects of limited sampling with respect to camera and projector appears undetectable. Therefore, the metrics relating to the probing errors should be used as a relative, unbiased representation of the influence of the specimen position within the measuring volume, rather than serve as an absolute comparison between measurement systems at different scale ranges. This was confirmed by the second experiment, in which the tooth height of a formed gear from SBMF was extracted and the entire surface was compared with a measurement from a laser scanning confocal microscope. In particular, the endoscopic sensor with a working distance of 20 mm (TR73 Endo 20), which was developed in conjunction with the TCRC 73, has been shown to deliver by far the best results. Particularly, the sensors operating in larger scale ranges can poorly reconstruct filigree structures with a high spatial frequency due to their lower metrological structural resolution.

Basically, however, it can be seen that regardless of the system considered, the examined form element can be measured with sufficient detail and resolution. Even with the ATOS GOM Compact 2M M2 with the largest measuring volume, the structure of the tooth tip can still be recognized, whereby the deviations are still small enough for an evaluation. The studies presented also show that a significant deviation from the reference geometries occurs, especially with the somewhat older LMI/GFM MikroCad pico sensor. This renders a comparison difficult, since more modern sensors generally have a higher performance.

One of the main applications for fringe projection profilometry in sheet-bulk metal forming is the ability to make statements about any varying process parameters in production on the final component geometry. For this purpose, it is essential to have suitable measuring systems available. In the long term, quality assurance mechanisms play a central role in the batch production of components manufactured using sheet-bulk metal forming operations. In addition to aspects, such as the time required for measurement, handling of the measurement system, and accessibility to measurement areas, the most accurate possible recording of the geometry is decisive. The development of existing systems or the comparison of different available instruments is, therefore, essential to provide this technology with the necessary tools. The findings developed in this research help to identify suitable sensors for different requirements. Thus, in comparison of all assessed 3D scanners, the use of the TR73 Endo 20 endoscope turned out to be the most target-oriented equipment for the reconstruction of a gear geometry with the given dimensions. It was observed that the applicability of the measuring systems could vary depending on the size and filigree of the investigated geometries. Thus, it would be grateful in later applications to measure more global characteristics, such as eccentricity or roundness of cylindrical bodies, even with less accurate systems, if mandatory quality criteria are sufficiently covered. Another aspect to be considered is the influence of the surface quality of the components on the quality of the measurement. In the current investigation, it was shown that this criterion has a decisive influence, especially for optical measuring systems, due to any reflective surfaces. Therefore, this aspect must be taken into account when designing measuring systems for subsequent SBMF processes.

For the *SLASSY* engineering workbench, the findings are integrated into in the knowledge base. *SLASSY* enables a *Design for Metrology* approach by offering product designers a preferred measurement method based on their actual part designs. This is to support product developers in sheet-bulk metal forming with the results and information of this contribution and supporting further investigations. Additionally, tools for optimal sensor selection to support the manufacturing and design process of the sheet-bulk metal forming components can be provided on the basis of the presented research. Optimal sensor positioning with regard to the measurement volume and a possible specimen part and inspected features, thus, can also be supported. These aspects need to be elaborated further.

## 9. Conclusions

The experiments presented in this study demonstrate that, even under ideal metrological conditions, the 3D scanners exhibit significantly non-uniform behavior depending on the position within the measuring volume. Therefore, the common approach of characterizing a 3D scanner, for instance, by means of maximum permissible deviations and tolerances, does not reflect the local characteristics of a optical, triangulation-based sensor. Due to the high number of measuring positions and the fine positioning grid, this study enables a precise assessment and analysis of the respective characteristics within the measuring volume.

In some areas, significantly higher accuracies can be achieved, while, at other positions in the measurement volume, the reconstruction is significantly inferior. However, it has also become apparent that the most accurate reconstruction of a geometric feature is not necessarily possible at the same position as the most uniform and low-noise surface reconstruction. A local comparison of the probing errors with respect to size and form clearly reveals the different characteristics of the examined measuring devices.

The actual shape and size of the respective measuring volume may also deviate significantly from the general specifications, as provided by some manufacturers. Under certain circumstances, the shape of the measured volume may not be represented by a cuboid or frustum. This is particularly the case when the influence of the field of curvature increases. Further differentiation with respect to the maximum reconstructable surface area has revealed a pronounced dependence in the direction of the optical axis for some sensors.

The conducted experiments provide the basis for an adapted measuring approach to enable the most accurate possible metrological analysis of various specimen parts. In conjunction with the featured engineering workbench, the sensor of the appropriate scale range can be identified and optimally positioned with respect to the given measurement application. Furthermore, this study allows position-dependent trade-offs to be made between the largest possible surface reconstruction, the optimal reconstruction of a geometric feature, and the most uniform surface possible.

In order to supplement the investigations by a more practical application and to deviate from ideal measurement conditions, as well as to investigate possible scale effects, the tooth of a sheet-bulk metal formed gear was examined in a second experiment. The advantage of sensors with a significantly smaller working distance and measuring volume could be observed, since finer geometric structures, such as neckings, were reconstructed more accurately. Sensors with coarser resolutions can, therefore, only reconstruct geometric features, such as tooth height, well. Therefore, it became apparent that the limitations of the metrological structure resolution due to the fixed lateral and axial resolution of each sensor could not be properly reflected in the first experiment, and further investigations and complementary experiments should follow this study.

## 10. Further Research

In order to provide absolute, comparable and generalizable statements regarding the expectable measurement deviations, it is suggested to conduct the experiments with different sized spheres. On the one hand, this should provide better comparable results, especially for sensors with smaller measurement volume and higher lateral and vertical resolution, and, at the same time, allow conclusions to be drawn about the optimal spatial frequency of each sensor. Since the current experiments were already expensive and time-consuming, and since calibrated spheres with similar optical cooperativity are not available in various diameters, a simple extension of the experiments does not appear to be appropriate. Since the sampling of the spatial frequency also depends strongly on the projected fringe frequencies, which are not always fixed for each measuring system, there is even a further degree of freedom, which must be taken into account for further experiments. It is, therefore, recommended to perform a separate experiment, which only investigates the influence of the sphere size and does not consider the relative position within the measuring volume. For the most systematic investigations possible, it is recommended to examine many different sphere diameters. It may be possible to print them using the stereolithography process or mill them using various ball mills. The resulting negative sphere standard should then be optically cooperatively coated and calibrated. Alternatively, conventional approaches could be used to identify the optimal spatial frequency at different reference standards [65]. The advantage of spherical geometries, however, is that the spatial structure resolution can related to the orientation within the measuring volume. By utilizing the experimental setup presented in this work, further investigations regarding the sensor pose, are conceivable. It is reasonable to expect that there will be some influence on the specimen pose in the measurement volume, relative to the triangulation base, affecting the optimal spatial frequency. The surface orientation relative to the sensor pose can also be assessed in further experiments, since a sphere, as used, in combination with a large number of measurement poses, can represent any possible surface normal at any position.

Despite the black box nature of commercial sensors, the influence of meshing and possible additional polygonal point sampling, as performed in Section 7, should be investigated in further experiments, as an influence on the quality of the reconstruction can be expected [66]. 

## Figures and Tables

**Table 1:** List and overview of the applied commercial fringe projection 3D scanners.

Name	Manufacturer	Measuring Volume	Resolution	
			Lateral	Axial
ATOS Core 200 5M	GOM GmbH (Braunschweig, Germany)	195 × 143 × 158 ${\mathrm{mm}}^{3}$	80 µm	80 µm
ATOS Compact Scan 2M		125 × 90 × 90 ${\mathrm{mm}}^{3}$	75 µm	75 µm
Measuring Range 1				
ATOS Compact Scan 2M		250 × 190 × 190 ${\mathrm{mm}}^{3}$	153 µm	153 µm
Measuring Range 2				
MicroCAD 1,0	LMI Technologies Inc.	13 × 10 × 3 ${\mathrm{mm}}^{3}$	17 µm	1 µm
	(Burnaby, BC, Canada)			
	Former: GF Messtechnik GmbH			
	(Teltow, Germany)			

**Table 2:** Parameters from the calibration certificate of the reference sphere.

Nominal Diameter	Actual Diameter	Roundness	Measurement Uncertainty	
			Diameter (k = 2)	Roundness
Ø 30 mm	Ø 29.9915 mm	0.7 µm	0.76 µm	0.5 µm

**Table 3:** Geometric dimensions of the experimentally determined measuring ranges of all applied measuring systems.

Sensor	Sphere Coverage		Bounding Box / mm			Volume /
	$A_{\mathrm{rel}}$	$A_{\mathrm{abs}}$	${\mathrm{BB}}_{X}$	${\mathrm{BB}}_{Y}$	${\mathrm{BB}}_{Z}$	${\mathrm{mm}}^{3}$
ATOS Compact Scan 2M MR 2	0.9	3.28 $\times \;10^{-1}$	193.48	174.93	175.09	4.98 $\times \;10^{6}$
	0.8	2.92 $\times \;10^{-1}$	200.19	187.65	181.61	5.65 $\times \;10^{6}$
	0.7	2.55 $\times \;10^{-1}$	205.79	195.07	182.70	5.94 $\times \;10^{6}$
ATOS Core 200 5M	0.9	2.10 $\times \;10^{-1}$	136.34	188.74	93.68	1.43 $\times \;10^{6}$
	0.8	1.87 $\times \;10^{-1}$	158.77	195.37	128.00	2.68 $\times \;10^{6}$
	0.7	1.64 $\times \;10^{-1}$	160.50	199.78	131.58	2.94 $\times \;10^{6}$
ATOS Compact Scan 2M MR 1	0.9	3.40 $\times \;10^{-1}$	72.75	97.89	84.83	4.71 $\times \;10^{5}$
	0.8	3.02 $\times \;10^{-1}$	80.26	104.42	94.87	6.04 $\times \;10^{5}$
	0.7	2.65 $\times \;10^{-1}$	83.54	107.98	96.37	6.56 $\times \;10^{5}$
TR 73 Endo 20	0.9	3.12 $\times \;10^{-2}$	13.96	11.39	4.47	2.96 $\times \;10^{2}$
	0.8	2.77 $\times \;10^{-2}$	18.42	16.86	6.81	1.08 $\times \;10^{3}$
	0.7	2.43 $\times \;10^{-2}$	19.88	18.98	7.87	1.50 $\times \;10^{3}$
LMI/GFM MikroCad pico	0.9	4.92 $\times \;10^{-2}$	7.96	10.45	2.46	8.77 $\times \;10^{1}$
	0.8	4.37 $\times \;10^{-2}$	10.89	14.49	4.29	3.25 $\times \;10^{2}$
	0.7	3.82 $\times \;10^{-2}$	11.64	18.05	4.84	4.92 $\times \;10^{2}$
TR 73 Endo 10	0.9	3.44 $\times \;10^{-3}$	8.32	6.88	2.57	3.87 $\times \;10^{1}$
	0.8	3.06 $\times \;10^{-3}$	13.73	12.30	3.55	2.07 $\times \;10^{2}$
	0.7	2.68 $\times \;10^{-3}$	16.54	17.83	4.53	5.05 $\times \;10^{2}$

## Data Availability

The data presented in this study are available on request from the corresponding author. The data are not publicly available due to very large data volumes. The data of this study will be stored for at least 5 years while the evaluation scripts will be available indefinitely.

## Abbreviations

The following abbreviations are used in this manuscript:

BB	Bounding box
CAD	Computer aided design
CLSM	Confocal laser scanning microscope
DOF	Depth of field
FEM	Finite element method
FPP	Fringe projection profilometry
HDR	High dynamic range
ICP	Iterative closest point (algorithm)
MR	Measuring range
NN	Nearest neighbor
SBMF	Sheet-bulk metal forming
SLASSY	Self-learning assistance system
TCRC	Transregional collaborative research center
WD	Working distance
